# Efficacy and safety of supraglottic jet oxygenation and ventilation to minimize sedation-related hypoxemia: a meta-analysis with GRADE approach

**DOI:** 10.1186/s13643-024-02707-w

**Published:** 2024-11-14

**Authors:** I-Wen Chen, Wei-Ting Wang, Pei-Chun Lai, Chun-Ning Ho, Chien-Ming Lin, Yao-Tsung Lin, Yen-Ta Huang, Kuo-Chuan Hung

**Affiliations:** 1 Department of Anesthesiology, Chi Mei Medical Center, Liouying, Tainan City, Taiwan; 2 Department of Anesthesiology, E-Da Hospital, I-Shou University, Kaohsiung City, Taiwan; 3 Education Center, National Cheng Kung University Hospital, College of Medicine, National Cheng Kung University, Tainan City, Taiwan; 4 Department of Paediatrics, College of Medicine, National Cheng Kung University Hospital, National Cheng Kung University, Tainan City, Taiwan; 5 Department of Anesthesiology, Chi Mei Medical Center, No. 901, ChungHwa Road, YungKung Dist, Tainan City, 71004 Taiwan; 6 School of Medicine, College of Medicine, National Sun Yat-Sen University, Kaohsiung City, Taiwan; 7 Department of Surgery, National Cheng Kung University Hospital, College of Medicine, National Cheng Kung University, Surgical Intensive Care Unit, No.138, Sheng Li Road, Tainan City, 704302 Taiwan

**Keywords:** Supraglottic jet oxygenation and ventilation, Hypoxemia, Sedation, Propofol, Wei nasal jet tube

## Abstract

**Introduction:**

Hypoxemia is a common complication of sedation. This meta-analysis aimed to evaluate the efficacy and safety of supraglottic jet oxygenation and ventilation (SJOV) in preventing hypoxemia during sedative procedures.

**Methods:**

Randomized controlled trials (RCTs) that compared SJOV with conventional oxygen therapy in sedated patients were searched in five databases (MEDLINE, EMBASE, Cochrane Library, China National Knowledge Infrastructure [CNKI], and Google Scholar) from their inception to March 2024. The primary outcome was the proportion of patients who developed hypoxia (SpO_2_ < 90%). The secondary outcomes included subclinical respiratory depression (90% ≤ SpO_2_ < 95%), severe hypoxemia (SpO_2_ < 75%), airway interventions, adverse events, hemodynamics, propofol dosage, and procedure time. The certainty of evidence was determined using the Grading of Recommendations Assessment, Development and Evaluation (GRADE) approach.

**Results:**

Twelve trials (*n* = 3058) were included in the analysis. The evidence suggests that SJOV results in a large reduction in the risk of hypoxemia (risk ratio [RR], 0.26; 95% confidence interval, 0.19–0.36; low certainty) and subclinical respiratory depression (RR, 0.40; low certainty) compared with the control. SJOV likely resulted in a large reduction in the risk of severe hypoxemia (RR, 0.22; moderate certainty). In addition, it may result in a large reduction in the need for jaw lift (RR, 0.22; low certainty) and mask ventilation (RR, 0.13; low certainty). The risk of sore throat probably increases with SJOV (RR, 1.71; moderate certainty), whereas SJOV may result in little to no difference in nasal bleeding (RR, 1.75; low certainty). Evidence is very uncertain regarding the effect of SJOV on hemodynamics (very low certainty) and procedure time (very low certainty). SJOV probably resulted in little to no difference in sedative doses between the groups (moderate certainty).

**Conclusion:**

According to the GRADE approach, SJOV likely results in a large reduction in the risk of severe hypoxemia but probably increases the risk of sore throat. Compared with the control, evidence suggests that SJOV results in a large reduction in the risk of hypoxemia, subclinical respiratory depression, and the need for airway manipulation, with little to no difference in nasal bleeding. The integration of SJOV into clinical practice may help minimize hypoxemic events in at-risk patients.

**Supplementary Information:**

The online version contains supplementary material available at 10.1186/s13643-024-02707-w.

## Introduction

Procedural sedation and analgesia are commonly performed to provide patient comfort and minimize pain, anxiety, and movement during invasive procedures [1–3]. In clinical settings, propofol is the preferred sedative because of its ability to induce sedation quickly and allow rapid recovery [4, 5]. However, the use of propofol with or without opioids can lead to dose-dependent respiratory depression and airway obstruction, thereby increasing the risk of hypoxemic events [6, 7]. Approximately 12–33% of patients experience transient oxygen desaturation during procedural sedation [6, 8–11]. Severe intraprocedural desaturation often requires brief positive-pressure ventilation to restore adequate oxygenation, which may interfere with the procedure. If untreated, these episodes of hypoxemia can progress to cyanosis, arrhythmia, organ dysfunction, and cardiovascular collapse [12–14]. In this regard, hypoxemia is responsible for as many as 25% of anesthesia-related deaths [15]. Minimizing sedation-related hypoventilation and maintaining optimal oxygenation throughout the procedure are critical [16]. Researchers have been interested in exploring alternative options to propofol [17–19] or combining other sedative agents with propofol [20–24] to improve patient safety during sedation. However, substantiating the efficacy and safety of these interventions is time-consuming.

In addition to improvements in pharmacological approaches, advanced oxygen delivery techniques such as high-flow nasal oxygen (HFNO) therapy have been reported to reduce the occurrence of hypoxemia during procedural sedation [25–27]. These advanced high-flow systems are often utilized to avert respiratory failure in critical care settings [28, 29], so their use in sedation settings remains uncommon. Supraglottic jet oxygenation and ventilation (SJOV) is an alternative technique that involves inserting a catheter (e.g., Wei nasal jet tube) into the nose or mouth, positioning its tip just above the vocal cords, and connecting it to a jet ventilator to deliver high-pressure pulses of oxygen [30, 31]. The high-pressure jet of oxygen helps to push oxygen into the lungs and flush out carbon dioxide to oxygenate the patient and provide ventilation [30]. SJOV can reduce the risk of hypoxemia in patients who undergo bronchoscopy or colonoscopy under sedation [32–34]. Although SJOV may be a promising technique for reducing the risk of hypoxemia, its efficacy and safety have not yet been assessed using a systematic approach. The number of diagnostic and therapeutic procedures requiring sedation has increased substantially [35, 36]. Consequently, ensuring the safety and quality of sedation is necessary to meet this increased demand. This systematic review and meta-analysis aimed to evaluate the existing evidence regarding the efficacy and safety of SJOV compared with standard oxygenation in minimizing sedation-related hypoxemia.

## Method

This systematic review and meta-analysis adhered to the Preferred Reporting Items for Systematic Reviews and Meta-Analyses (PRISMA) guidelines. The protocol for this meta-analysis was registered with the International Prospective Register of Systematic Reviews (PROSPERO) prior to conducting the literature search and data extraction (registration number: CRD42024519442; registration date: March 2, 2024).

### Search strategy and data sources

A literature search was conducted to identify all relevant randomized controlled trials (RCTs) on the efficacy and safety of SJOV in minimizing sedation-related hypoxia. The following databases were searched from inception to March 2024: MEDLINE, EMBASE, Cochrane Central Register of Controlled Trials (CENTRAL), China National Knowledge Infrastructure (CNKI), and Google Scholar. The search strategy combined controlled vocabulary specific to each database (e.g., Medical Subject Headings terms in MEDLINE) and free-text words relevant to the research topic. Search terms included (“Supraglottic jet oxygenation” or “SJOV” or “Wei nasal jet tube” or “supraglottic jet oxygenation and ventilation” or “Transnasal jet ventilation” or “Wei nasal jet ventilation” or “jet ventilation” or “apneic ventilation” or “high frequency jet ventilation”) AND (“Sedation” or “Sedative procedure” or “Procedural sedation” or “Monitored anesthesia care” or “Colonoscopy” or “Bronchoscopy” or “Endoscopic retrograde cholangiopancreatography” or “gastrointestinal endoscopy” or “gastroscopy” or “propofol” or “intravenous anesthesia”) AND (“Respiratory depression” or “Hypoxaemia” or “Nasal bleeding” or “Hypoxia” or “Adverse events” or “tracheal intubation”). No restrictions were placed on language or publication year. The reference lists of all eligible studies and relevant systematic reviews were manually examined to identify additional relevant studies. The search strategy for one of the databases is listed in Supplemental Table 1.

### Inclusion and exclusion criteria

The following inclusion criteria were used: (1) patients aged ≥ 18 years undergoing procedures under sedation with or without analgesia; (2) intervention involving SJOV administered through an oral or nasal route; (3) control group using conventional oxygenation techniques, such as oxygen supplementation via nasal cannula; (4) reported incidence of hypoxemia and/or adverse events; and (5) RCTs with full text available.

Studies were excluded if (1) they were animal or simulation studies; (2) they focused on patients under general anesthesia with or without muscle relaxants; (3) SJOV was employed either before or during tracheal intubation; (4) they were presented as case reports, observational studies, review articles, case series, or conference abstracts; or (5) a tracheal tube or laryngeal mask airway was used to protect the airway during sedation. Two reviewers independently assessed all studies for eligibility using predefined criteria. We excluded conference abstracts from our analysis because they often lack detailed methodology and results, making it difficult to assess study quality and risk of bias. In addition, data published in conference abstracts are often preliminary and may change significantly after peer review, risking inaccuracies in the meta-analysis.

### Selection process for studies

The study selection process was conducted in two phases. In the first phase, two independent reviewers screened the titles and abstracts of the retrieved records to identify potentially eligible studies. In the second phase, the same reviewers assessed the full texts of potentially eligible articles in detail using the same eligibility criteria. Disagreements at both stages were resolved by consensus or consultation with a third reviewer.

### Data collection

Two independent reviewers extracted relevant data from the included studies in a standard form. The extracted information included the following:

1. Study characteristics: authors, year, study design, setting, and country.
2. Patient characteristics: demographics, American Society of Anesthesiologists’(ASA) Physical Status, sex distribution, and body mass index (BMI).
3. Details on intervention and control groups: device used and oxygen flow rate.
4. Details on the procedure: type of procedure performed, procedural timing, and dosage of propofol.
5. Outcomes: incidence of hypoxemia (as defined by the study), need for airway assistance (e.g., mask ventilation), adverse events (e.g., sore throat, nasal bleeding), and hemodynamic instability (e.g., hypertension).

For studies with multiple publications, the most complete and recent reports on outcomes were used. Missing data were requested by the authors via email. The extracted data were cross-checked by two reviewers to resolve any discrepancies.

### Outcome and definition

The primary outcome was the incidence of intraprocedural hypoxemia, defined as the proportion of patients experiencing < 90% oxygen desaturation during the procedure in each study group. The secondary outcomes included the incidence of subclinical respiratory depression (i.e., 90% ≤ SpO_2_ < 95%), severe hypoxemia (i.e., Spo2 < 75%), need for airway assistance (e.g., mask ventilation), hemodynamic instability, adverse events, and differences in propofol dosage and procedure time. Our definitions of hypoxemia and categorizations of hypoxemia severity were based on consistent criteria used across all included studies. Regarding procedural time, we considered a difference of at least 5 min to be clinically relevant based on clinical judgment.

### Quality of assessment for studies

The methodological quality of the included RCTs was assessed using the revised Cochrane risk of bias tool for randomized trials (RoB 2) [37]. Two independent reviewers assessed the presence of bias across the following domains: bias arising from the randomization process, bias due to deviations from the intended interventions, bias from missing outcome data, bias in the measurement of the outcome, and bias in the selection of the reported result. For each domain, the risk of bias was categorized as low, “some concerns,” or high. An overall risk of bias judgment was made across the domains for each included trial. Disagreements between the two reviewers were resolved by discussion and consensus or by consulting a third reviewer. The results were drawn using the risk of bias visualization tool [38].

### Certainty of evidence

The certainty of evidence for each outcome was determined using the Grading of Recommendations Assessment, Development, and Evaluation (GRADE) approach [39]. Two independent reviewers rated the certainty of each outcome as high, moderate, low, or very low based on the risk of bias, inconsistency, indirectness, imprecision, and publication bias. The level of certainty within the domain of imprecision was adjusted downwards according to newly established criteria [40]. Any discrepancies in the GRADE assessments were discussed between the two reviewers to reach a consensus or resolved by a third author. GRADE certainty ratings were detailed, summarized into a table, and employed to determine the strength of evidence for guiding practice recommendations using the GRADEpro Guideline Development Tool (McMaster University and Evidence Prime, 2022; available at gradepro.org). In addition, we present the findings of the meta-analysis using a standardized approach to effectively communicate the results [41].

### Statistical analysis

All analyses were conducted using the Cochrane Review Manager (RevMan 5.3; Copenhagen: The Nordic Cochrane Center, The Cochrane Collaboration, 2014) or Comprehensive Meta-Analysis version 4 (Biostat, Englewood, NJ, USA). Dichotomous outcomes are expressed as risk ratios (RR) with 95% confidence intervals (CIs). Continuous outcomes were summarized using mean differences with 95% CIs. For studies with more than two control arms, participant data in the intervention group were split to create multiple pairwise comparisons while preventing double counting [42]. The number of events and participants in the shared study arms were evenly divided among the comparison groups. This approach enabled the inclusion of multiple control groups per study while avoiding overlap in participants across comparisons.

As clinical and methodological variability was expected between studies, a random-effects model was used for analysis, regardless of heterogeneity. Sensitivity analyses using the leave-one-out approach were conducted to evaluate the robustness of the findings. If at least 10 studies or datasets were included for any outcome, publication bias was assessed by visual inspection of funnel plots and Egger’s regression test for asymmetry. The I^2^ statistic was used to evaluate between-study heterogeneity for each outcome, with I^2^ > 75% indicating substantial heterogeneity. Sources of heterogeneity were explored through subgroup and meta-regression analyses, if substantial heterogeneity was present. A two-sided *p*-value < 0.05 was considered statistically significant for all analyses. Trial sequential analysis (TSA) was performed on the primary outcome to examine the robustness of the evidence. We applied a type I error of 5%, power of 80%, and relative risk reduction of 20% based on the minimal clinically important difference for intervention efficacy.

## Results

### Search results and study characteristics

**Fig. 1:**
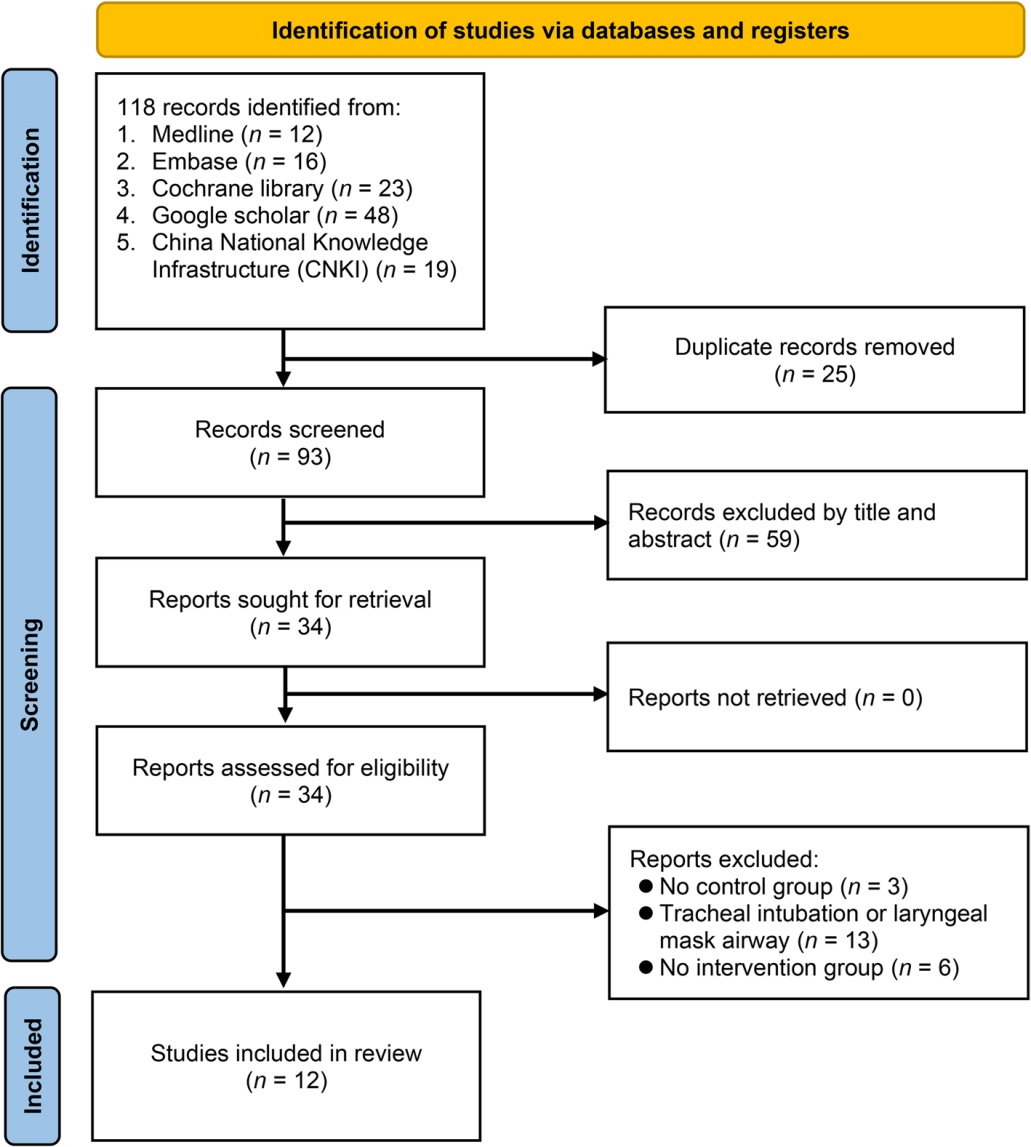
Flow chart of study selection

The initial literature search yielded 118 records from various databases, which was reduced to 93 after removing 25 duplicates (Fig. 1). Screening of titles and abstracts excluded 59 records that did not meet the inclusion criteria. Of the 34 full texts assessed, 22 were excluded for the following reasons: lack of control groups (*n* = 3), tracheal intubation or laryngeal mask airway involvement (*n* = 13), and absence of an intervention group (*n* = 6) (Supplemental Table 2). Ultimately, 12 RCTs (two arms: eight trials [32, 34, 43–48], three arms: four trials [31, 33, 49, 50]) with 3058 participants were included in the analysis (Table 1).

**Table 1:** Characteristics of 12 randomized controlled studies (*n* = 3058)

Studies	RCT design	Age (years)^⁋^	BMI (kg/m^2^)	Male (%)^a^	*N*	ASA	Sedation level	Sedation agents	SJOV setting (DP)^b^	Control setting (device/flow)	Procedure	Country
Fu 2023	2-arm	45/44	22/22	4846/	100	I–III	Deep sedation	Pro/re	18–20 psi	NPA 6 L/min	ERCP	China
Jiang 2022	2-arm	49/50	24/ 23	50/53	72	I–III	OAA/S: 1–2	Pro	15 psi	NC 2 L/min	GI endoscopy	China
Li 2019	2-arm	73/73	19/19	63/60	60	I–II	BIS < 60	Pro/Suf	35–45 psi	NC 5L/min	FB	China
Liang 2019 (1)	2-arm	45/43	24/23	0	120	I–II	BIS: 45–60	pro/re	14.5–43.5 psi	Mask 6 L/min	Hysteroscopy	China
Liang 2019 (2)	3-arm	44/45/44	33/33/33	0	100	I–II	BIS: 45–60	pro/re	14.5 psi	Mask 6 L/min WNJ 6 L/min	Hysteroscopy	China
Qin 2017	3-arm	47/46/48	23/23/23	47/45/46	1781	I–III	OAA/S: 2–3	pro	15 psi	NC 2 L/min, WNJ 2 L/min	GI endoscopy	China
Su 2024	3-arm	56/56/56	23/23/22	45/48/39	167	I–III	BIS 45–60	pro/re/dex	15 psi	NPA 4–6 L/min WNJ 4–6 L/min	ERCP	China
Wei 2024	3-arm	59/59/62	24/23/23	57/50/61	132	I–III	MOAA/S: 1–2	pro/re	15 psi	NC 4 L/min WNJ 4 L/min	FB	China
Wu 2020	2-arm	44/44	na	48/44	100	I–III	Deep sedation	pro	15 psi	NC 6L/min	gastroscopy	China
Yang 2023	2-arm	51/51	23/22	66/64	105	I–III	RSS score: 4–5	pro/re	14.5 psi	Mask	FB	China
Yang 2016	2-arm	41/43	22/22	36/13	49	I–II	MAC	Pro/fe	15 psi^c^	NC 6 L/min	Colonoscopy	China
Zha 2021	2-arm	52/53	21/21	77/77	272	I–III	MOAA/S 2–3	pro/re	15 psi	NC 4 L/min	FB	China

*ASA* Anesthesiologists physical status classification system, *BIS* Bispectral index, *BMI* Body mass index, *dex* dexmedetomidine, *DP* Driving pressure, *ERCP* Endoscopic retrograde cholangiopancreatography, *fe* fentanyl, *FB* Flexible bronchoscopy, *GI* Gastrointestinal, *MAC* Monitored anesthesia care, *MOAA/S* Modified Observer Assessment of Alertness/Sedation Scale, *na* not available, *NC* Nasal cannula, *NPA* nasopharyngeal airway, *OAA/S* Observer’s Assessment of Alertness/Sedation, *pro* Propofol, *RCT* Randomized controlled trial, *re* remifentanil, *RSS* Ramsay sedation scale, *SJOV* Supraglottic jet oxygenation and ventilation, *suf* sufentanil, *WNJ* Wei nasal jet tube

^a^presented as intervention/control groups

^b^frequency: 8–20 min, FiO_2_ = 1

^c^Cook tube exchanger used

The mean age of the participants ranged from 41 to 73 years, with the percentage of males ranging from 0 to 77%. Eleven RCTs included the general population, whereas one trial focused on obese patients [49]. Most studies enrolled patients with ASA physical status I–III who received sedative agents, including propofol, with or without opioids. Sedation levels varied across studies, ranging from monitoring anesthesia care to deep sedation. SJOV was delivered using a Wei nasal jet tube in 11 trials and via an 11 Fr tube exchanger in one trial [34]. In all cases, the devices were inserted through the nostrils. The SJOV settings included an oxygen flow rate of 8–20 L/min and driving pressure of 14.5–45 psi, with a commonly used driving pressure of 15 psi. The control group received oxygen supplementation at 2–6 L/min, using various airway devices. Among the five studies that examined CO_2_ levels with SJOV use, three found that the levels were associated with a low risk of CO_2_ retention, whereas two did not report similar findings (Supplemental Table 3). Additionally, two studies measured gastric volume using ultrasound before and after SJOV use, and the results showed no significant difference in gastric volume (Supplemental Table 3). No studies reported the occurrence of barotrauma (Supplemental Table 3). All studies were conducted in China. The funding sources for each study are provided in Supplemental Table 4.

### Risk of bias

**Fig. 2:**
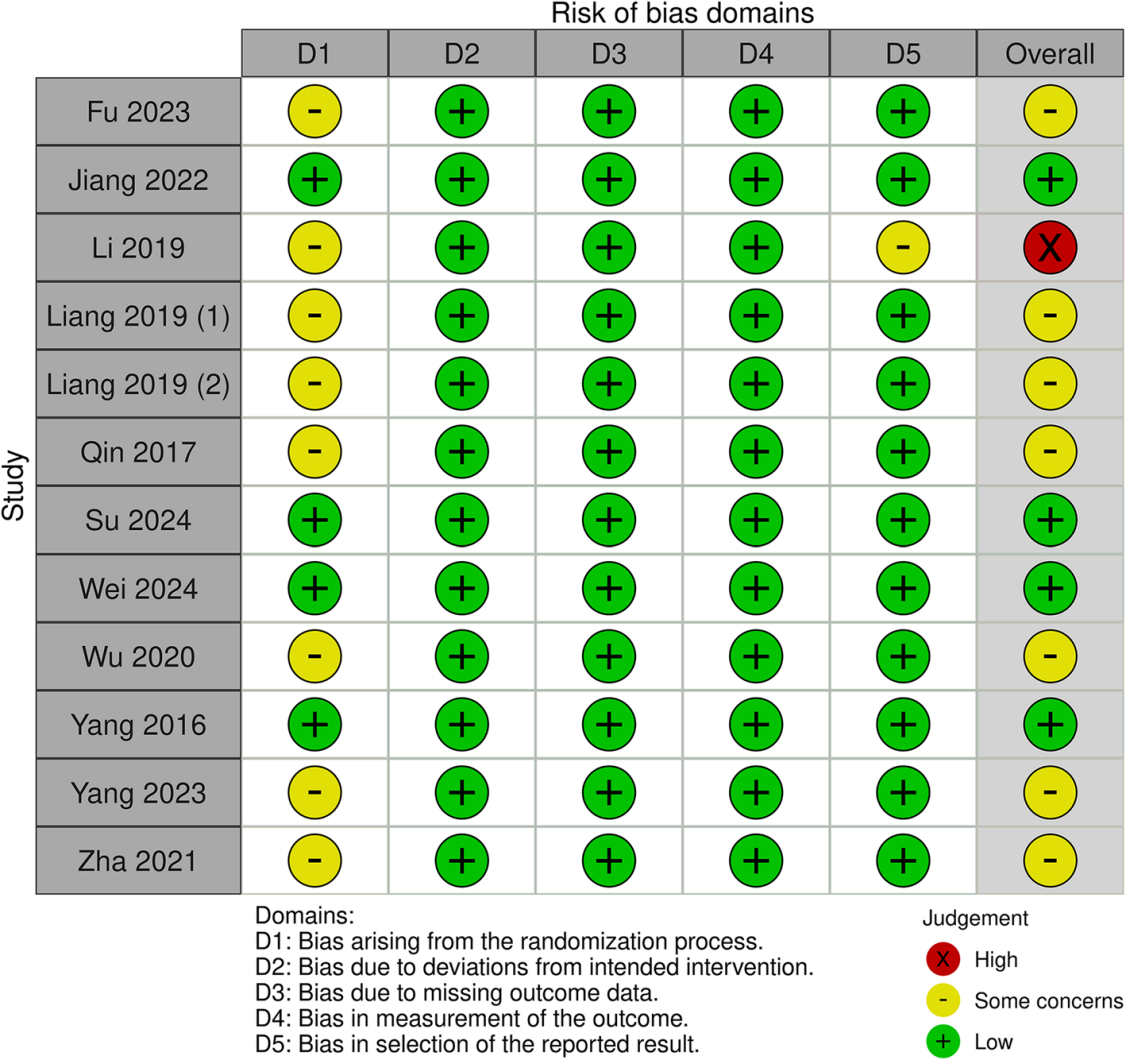
Risk of bias assessment of included studies by using the revised Cochrane risk-of-bias tool for randomized trials (RoB 2)

The risk of bias assessment of the included studies for the primary outcome is shown in Fig. 2. The majority of trials were judged to have some concern in the randomization process, with the exception of four studies that had a low risk of bias. All studies were considered to have a low risk of bias for deviations from the intended interventions, missing outcome data, and outcome measurements. Regarding the risk of bias in the selection of reported results, most studies were judged to have a low risk, except for one study that had some concerns. The overall risk of bias was low in four studies [33, 34, 44, 50], while seven studies had some concerns [31, 43, 45–49]. One of the studies had a high risk of bias [32]. The risk of bias assessment for the secondary outcomes is presented in Supplemental Table 5.

### Outcomes

#### Primary outcome: risk of hypoxemia

**Fig. 3:**
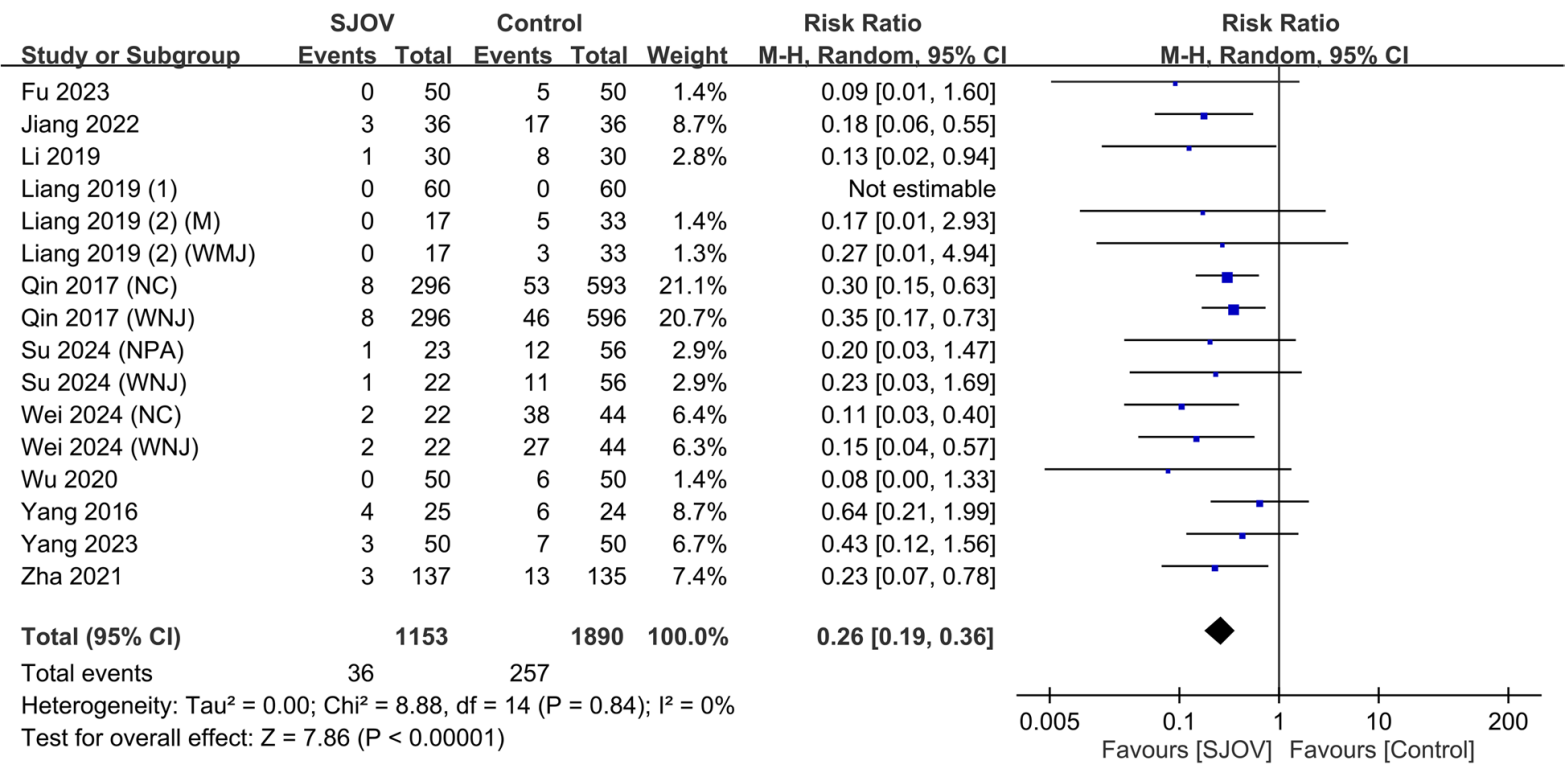
Forest plot showing the efficacy of supraglottic jet oxygenation and ventilation (SJOV) against intraprocedural hypoxemia defined as SpO_2_ < 90%. CI: confidence interval. M-H, Mantel–Haenszel; NPA, nasopharyngeal airway; WNJ, Wei nasal jet tube; NC, nasal cannula; M, mask

**Fig. 4:**
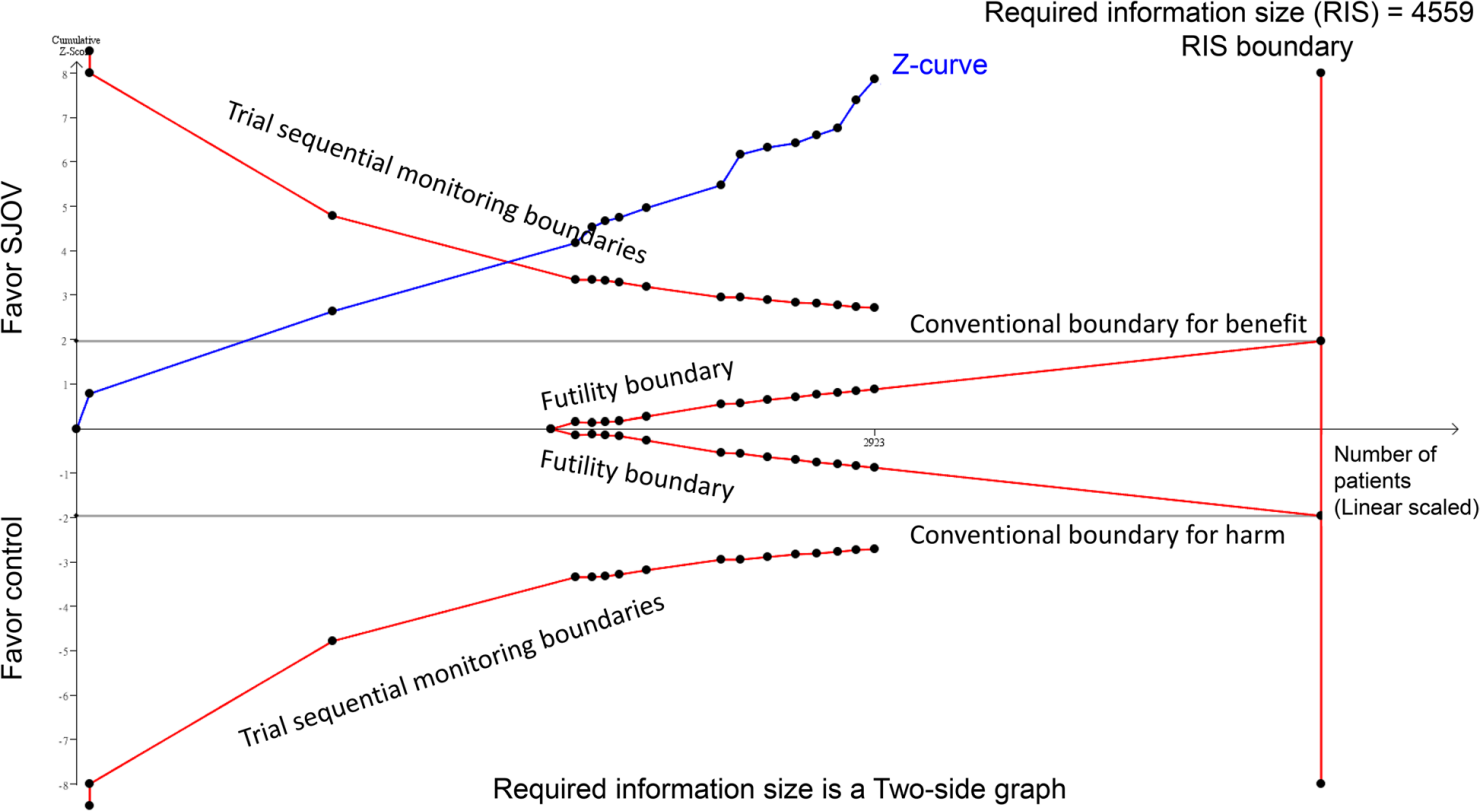
Trial sequential analysis (TSA) of the primary outcome demonstrated that the z-curve crossed the trial sequential monitoring boundaries, indicating the strong robustness of the evidence

The evidence suggested that SJOV results in a large reduction in the risk of intraprocedural hypoxemia (SpO_2_ < 90%) compared to the conventional oxygenation techniques (3.1% vs. 13.6%; RR = 0.26, 95% CI 0.19 to 0.36; *p* < 0.00001; *I*^2^ = 0%, low certainty evidence) (Fig. 3). Subgroup or meta-regression analyses were not performed because of lack of heterogeneity. TSA showed that the z-curve crossed trial sequential monitoring boundaries, indicating the robustness of the evidence (Fig. 4).

#### Secondary outcomes

**Fig. 5:**
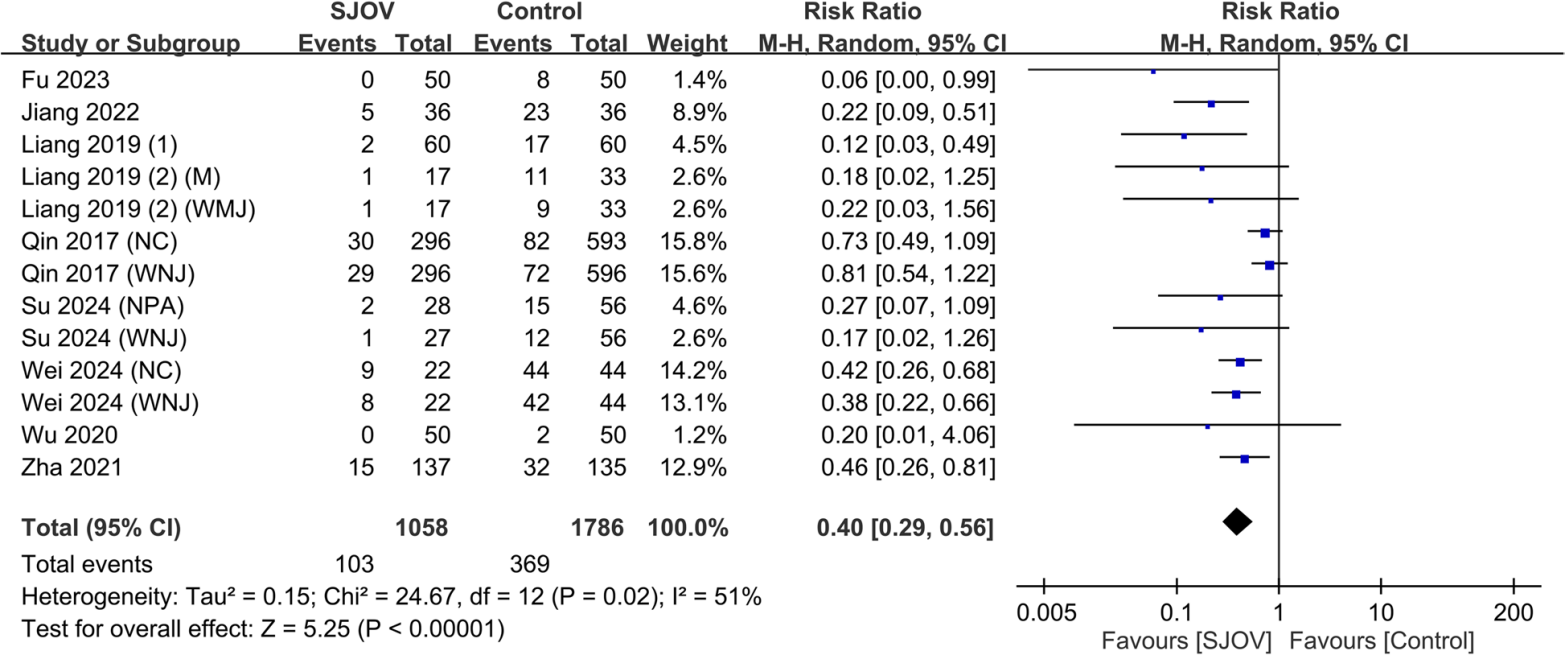
Forest plot showing the risk of subclinical respiratory depression in the supraglottic jet oxygenation and ventilation (SJOV) versus control group. CI, confidence interval; M-H, Mantel–Haenszel; NPA, nasopharyngeal airway; WNJ, Wei nasal jet tube; NC, nasal cannula; M, mask

**Fig. 6:**
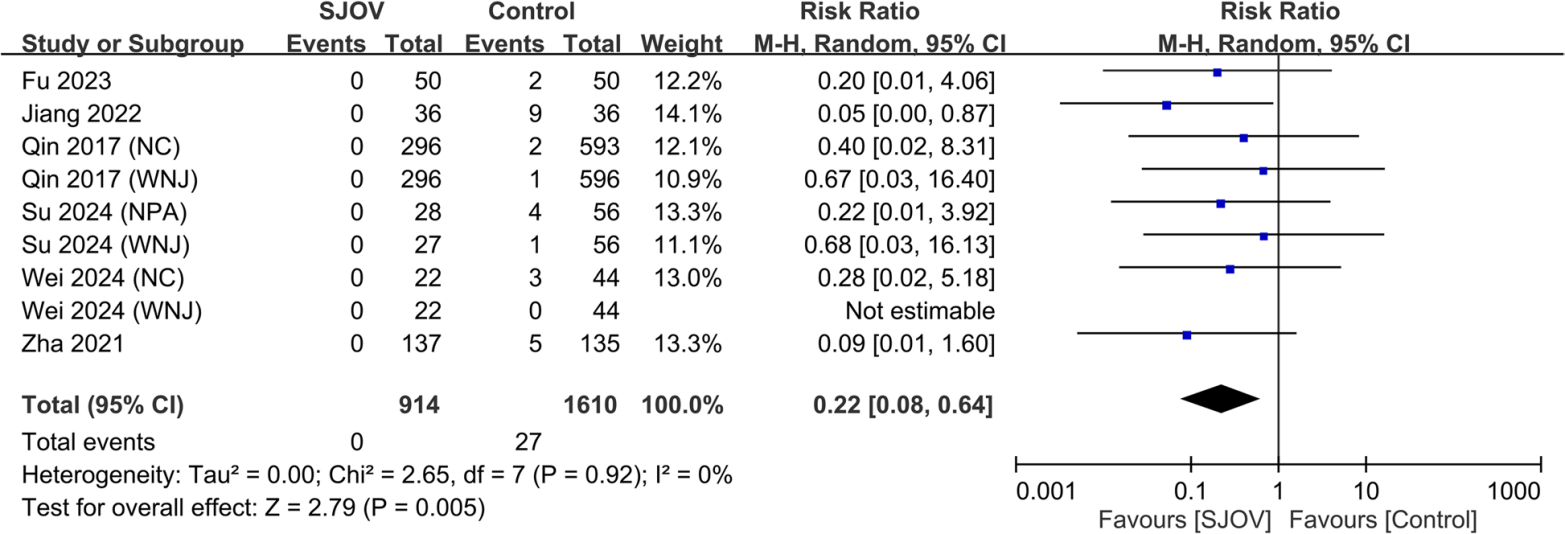
Forest plot showing the risk of severe hypoxemia in the supraglottic jet oxygenation and ventilation (SJOV) versus control group. CI, confidence interval; M-H, Mantel–Haenszel; NPA, nasopharyngeal airway; WNJ, Wei nasal jet tube; NC, nasal cannula

**Fig. 7:**
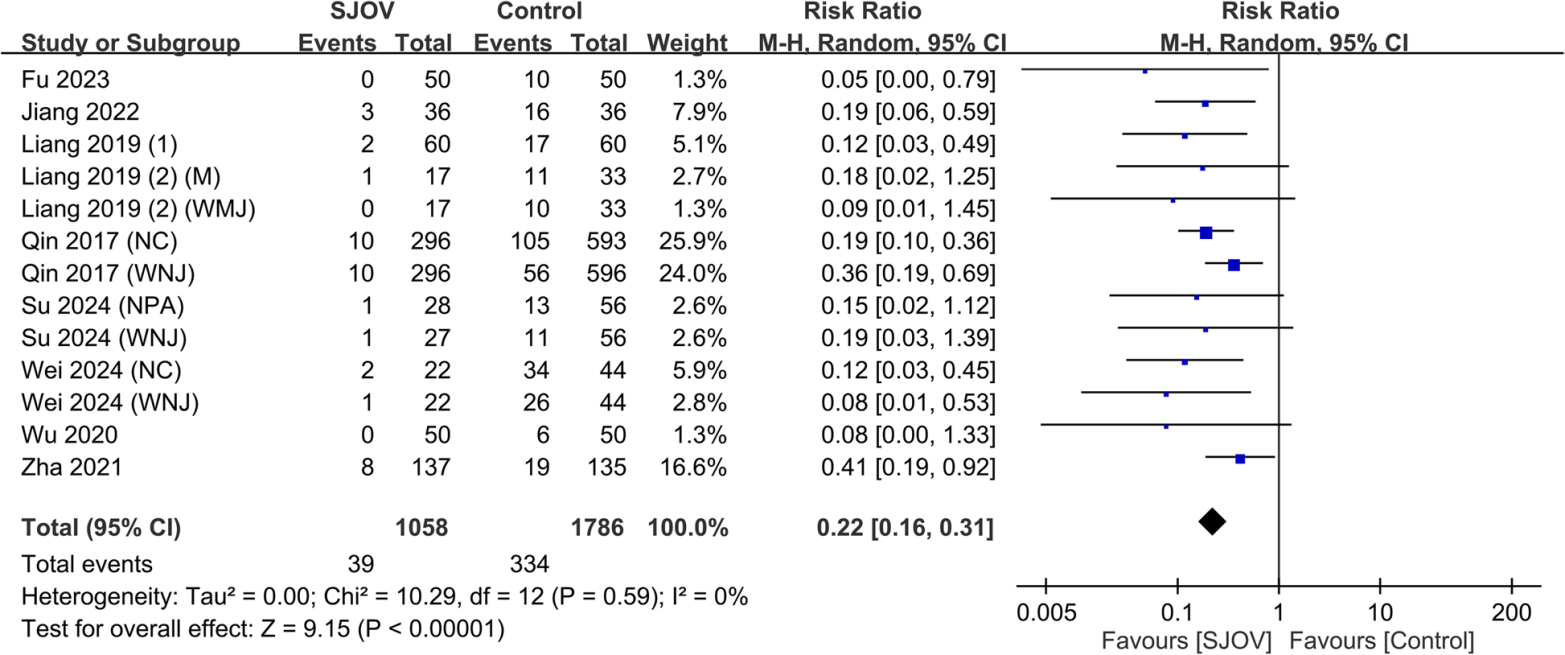
Forest plot showing the risk of jaw lift in the supraglottic jet oxygenation and ventilation (SJOV) versus the control group. CI, confidence interval; M-H, Mantel–Haenszel; NPA, nasopharyngeal airway; WNJ, Wei nasal jet tube; NC, nasal cannula; M, mask

**Fig. 8:**
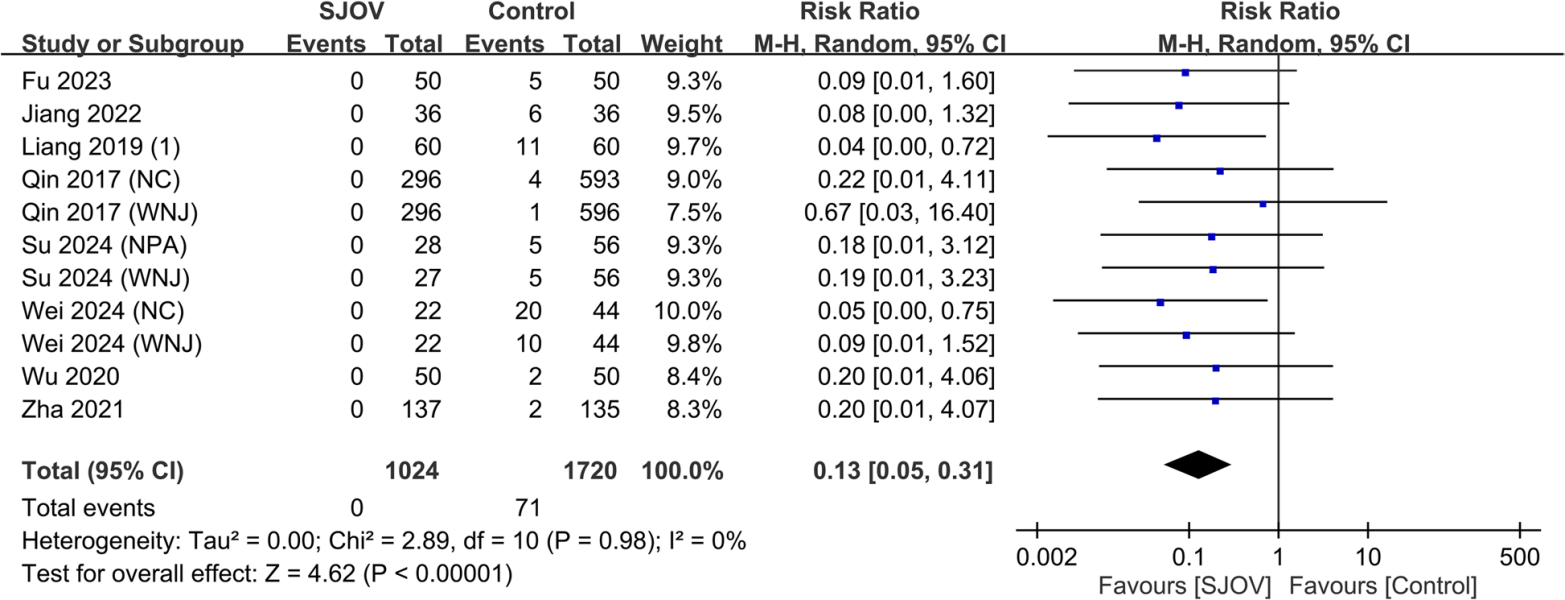
Forest plot showing the risk of mask ventilation in the supraglottic jet oxygenation and ventilation (SJOV) versus control group. CI, confidence interval; M-H, Mantel–Haenszel; NPA, nasopharyngeal airway; WNJ, Wei nasal jet tube; NC, nasal cannula

The evidence suggested that SJOV results in a large reduction in the risk of subclinical respiratory depression (SpO_2_ 90–95%) (9.7% vs. 20.7%, RR = 0.40, 95% CI 0.29–0.56, *p* < 0.00001; *I*^2^ = 51%, low certainty evidence) (Fig. 5). SJOV likely results in a large reduction in the risk of severe hypoxemia (SpO_2_ < 75%) (0 vs. 1.7%, RR = 0.22, 95% CI 0.08–0.64, *p* = 0.005; *I*^2^ = 0%, moderate certainty evidence) (Fig. 6). In addition, SJOV may result in a large reduction in the need for jaw lift (3.7% vs. 18.7%, RR = 0.22, 95% CI 0.16 to 0.31, *p* < 0.00001; *I*^2^ = 0%, low certainty evidence) (Fig. 7) and mask ventilation (0.0% vs. 4.1%, RR = 0.13, 95% CI 0.05 to 0.31, *p* < 0.00001; *I*^2^ = 0%, low certainty evidence) (Fig. 8). SJOV may result in little to no difference in nasal bleeding (2.1% vs. 1.6%, RR = 1.75, 95% CI 0.89–3.45, *p* = 0.11; *I*^2^ = 0%, low certainty evidence) (Supplemental Fig. 1). However, the risk of sore throat is probably increased with SJOV (6.7% vs. 4.7%, RR = 1.71, 95% CI 1.22–2.39, *p* = 0.002; *I*^2^ = 0%, moderate certainty evidence) (Supplemental Fig. 2).

The evidence is very uncertain regarding the effect of SJOV on the incidence rates of bradycardia (3.5% vs. 3.7%, RR = 0.82, 95% CI 0.52–1.28, *p* = 0.38; *I*^2^ = 0%, very low certainty evidence) (Supplemental Fig. 3), tachycardia (0.9% vs. 1.5%, RR = 0.74, 95% CI 0.33–1.68, *p* = 0.92; *I*^2^ = 4%, very low certainty evidence) (Supplemental Fig. 4), hypertension (0.8% vs. 2.0%, RR = 0.48, 95% CI 0.2–1.14, *p* = 0.1; *I*^2^ = 0%, very low certainty evidence) (Supplemental Fig. 5), and hypotension (2.7% vs. 2.7%, RR = 0.91, 95% CI 0.52–1.59, *p* = 0.74; *I*^2^ = 0%, very low certainty evidence) (Supplemental Fig. 6). SJOV probably resulted in little to no difference in sedative doses between the groups (MD = − 0.03 mg, 95% CI − 2.39 to 2.33, *p* = 0.98; *I*^2^ = 0%, moderate certainty evidence) (Supplemental Fig. 7). The evidence is very uncertain regarding the effect of SJOV on procedure time (mean difference − 0.6 min, 95% CI − 1.04 to − 0.16, *p* = 0.007; *I*^2^ = 81%, very low certainty evidence) (Supplemental Fig. 8). Although this difference was statistically significant, it is well below the minimal clinically important difference of 5 min and therefore may not be clinically important.

#### Sensitivity analysis

Sensitivity analyses using the leave-one-out method showed consistent results for most outcomes except for nasal bleeding, hypertension, and procedure time. After excluding one study [33], a higher nasal bleeding risk was found in the SJOV group (the *p*-value changed from 0.11 to 0.03). Removing one study [44] showed reduced hypertension risk in the SJOV group (*p*-value changed from 0.1 to 0.03). Excluding another study [32] indicated similar procedure times between groups (*p*-value changed from 0.007 to 0.21). These findings suggest inconsistencies in the effects of SJOV on nasal bleeding, hypertension, and procedure time.

#### Publication *bias*

An examination of the funnel plot for outcomes with more than 10 datasets was conducted, focusing on the risks of hypoxemia (Supplemental Fig. 9) (Egger’s test: *p* = 0.05), subclinical respiratory depression (Supplemental Fig. 10) (Egger’s test: *p* = 0.001), jaw lift (Supplemental Fig. 11) (Egger’s test: *p* = 0.007), mask ventilation (Supplemental Fig. 12) (Egger’s test: *p* = 0.0006), nasal bleeding (Supplemental Fig. 13) (Egger’s test: *p* = 0.51), sore throat (Supplemental Fig. 14) (Egger’s test: *p* = 0.18), dosage of propofol (Supplemental Fig. 15) (Egger’s test: *p* = 0.4), and procedure time (Supplemental Fig. 16) (Egger’s test: *p* = 0.01). The results based on Egger’s test indicated that publication bias was present for several outcomes, including subclinical respiratory depression, jaw lift, mask ventilation, and procedure time. The funnel plot also demonstrated asymmetry in these outcomes.

#### Certainty of evidence

The certainty of evidence for each outcome is summarized in Table 2. Evidence certainty received a very low grading for bradycardia, tachycardia, hypertension, hypotension, and procedure time. It was graded as low for another five outcomes, which included hypoxemia, subclinical respiratory depression, jaw–thrust, mask ventilation, and nasal bleeding, and as moderate for three outcomes: severe hypoxia, sore throat, and propofol dosage.

**Table 2:** The certainty of evidence for each outcome based on the Grading of Recommendations Assessment, Development and Evaluation (GRADE) approach

No. of studies	Outcomes	Certainty assessment					No. of patients		Effect		Certainty
		**A**	**B**	**C**	**D**	**E**	**SJOV**	**Control**	**Relative (95% CI)**	**Absolute (95% CI)**	
16	Hypoxemia	VS^a^	NS	NS	NS	None	36/1153 (3.1%)	257/1890 (13.6%)	RR 0.26 (0.19 to 0.36)	101 fewer per 1000 (from 110 to 87 fewer)	⨁⨁◯◯ Low
13	Subclinical respiratory depression	S^b^	NS	NS	NS	Y^c^	103/1058 (9.7%)	369/1786 (20.7%)	RR 0.40 (0.29 to 0.56)	118 fewer per 1000 (from 140 to 87 fewer)	⨁⨁◯◯ Low
9	Severe hypoxemia	S^b^	NS	NS	NS	None	0/914 (0.0%)	27/1610 (1.7%)	RR 0.22 (0.08 to 0.64)	13 fewer per 1000 (from 15 to 6 fewer)	⨁⨁⨁◯ Moderate
13	Jaw-thrust	S^b^	NS	NS	NS	Y^c^	39/1058 (3.7%)	334/1786 (18.7%)	RR 0.22 (0.16 to 0.31)	146 fewer per 1000 (from 157 to 129 fewer)	⨁⨁◯◯ Low
11	Mask ventilation	S^b^	NS	NS	NS	Y^c^	0/1024 (0.0%)	71/1720 (4.1%)	RR 0.13 (0.05 to 0.31)	36 fewer per 1000 (from 39 to 28 fewer)	⨁⨁◯◯ Low
12	Nasal bleeding	S^b^	NS	NS	S^d^	None	21/1023 (2.1%)	19/1157 (1.6%)	RR 1.75 (0.89 to 3.45)	12 more per 1000 (from 2 fewer to 40 more)	⨁⨁◯◯ Low
11	Sore throat	S^b^	NS	NS	NS	None	65/973 (6.7%)	52/1107 (4.7%)	RR 1.71 (1.22 to 2.39)	33 more per 1000 (from 10 to 65 more)	⨁⨁⨁◯ Moderate
8	Bradycardia	VS^a^	NS	NS	S^d^	None	26/747 (3.5%)	53/1433 (3.7%)	RR 0.82 (0.52 to 1.28)	7 fewer per 1000 (from 18 fewer to 10 more)	⨁◯◯◯ Very low
8	Tachycardia	VS^a^	NS	NS	S^d^	None	7/747 (0.9%)	22/1433 (1.5%)	RR 0.74 (0.33 to 1.68)	4 fewer per 1000 (from 10 fewer to 10 more)	⨁◯◯◯ Very low
8	Hypertension	VS^a^	NS	NS	S^d^	None	6/747 (0.8%)	29/1433 (2.0%)	RR 0.48 (0.20 to 1.14)	11 fewer per 1,000 (from 16 fewer to 3 more)	⨁◯◯◯ Very low
8	Hypotension	VS^a^	NS	NS	S^d^	None	20/747 (2.7%)	38/1433 (2.7%)	RR 0.91 (0.52 to 1.59)	2 fewer per 1,000 (from 13 fewer to 16 more)	⨁◯◯◯ Very low
12	Propofol dosage	S^b^	NS	NS	NS	None			-	MD 0.03 mg fewer (2.39 fewer to 2.33 more)	⨁⨁⨁◯ Moderate
12	Procedure time	VS^a^	S^e^	NS	NS	Y^c^			-	MD 0.6 min lower (1.04 lower to 0.16 lower)	⨁◯◯◯ Very low

A risk of bias, B Inconsistency, C Indirectness, D Imprecision, E other consideration, Y publication bias strongly suspected

*CI* Confidence interval, *MD* Mean difference, *RR* Risk ratio, *SJOV* Supraglottic jet oxygenation and ventilation, *VS* Very serious, *S* Serious, *NS* Not serious

Explanations:

^a^More than half of the enrolled studies were evaluated as having an overall risk of bias rated as “some concern,” and there was also one enrolled study assessed with a “high” overall risk of bias

^b^Over one-quarter of the enrolled studies were evaluated as having “some concern” regarding their overall risk of bias

^c^Egger’s test, *p* < 0.05

^d^A wide 95% confidence interval that crosses the minimally important difference threshold, set as 0.8 to 1.2

^e^I-square more than 60%

## Discussion

This meta-analysis of 12 RCTs (*n* = 3058) demonstrated that SJOV likely results in a large reduction in the risk of severe hypoxemia (RR, 0.22; moderate certainty) and probably results in little to no difference in sedative doses between the groups (moderate certainty). Additionally, the risk of sore throat is probably increased with SJOV (RR, 1.71; moderate certainty). The evidence also suggested that SJOV results in a large reduction in the risk of hypoxemia (RR, 0.26; low certainty), subclinical respiratory depression (RR, 0.40; low certainty), the need for jaw lift (RR, 0.22; low certainty), and mask ventilation (RR, 0.13; low certainty). Furthermore, SJOV may result in little to no difference in nasal bleeding (RR, 1.75; low certainty). Finally, evidence regarding the effect of SJOV on hemodynamics and procedure time is very uncertain (very low certainty).

SJOV is an innovative, minimally invasive technique that optimizes oxygenation by delivering high-flow, high-concentration oxygen through a specialized nasal tube or catheter [30]. The device is strategically positioned to direct a jet of oxygen towards the vocal cords, enabling rapid pulsatile delivery into the trachea [30]. This targeted approach enhances gas exchange and may increase lung functional residual capacity, thereby improving overall oxygenation [30]. SJOV can be used as a continuous oxygen source or integrated into a jet ventilator to provide oxygenation and ventilation support. The precise physiological mechanisms underlying the effect of SJOV on pulmonary shunt fraction and ventilation-perfusion matching remain to be fully elucidated [30]. SJOV offers a promising means of minimizing hypoxemia risk during procedural sedation by maintaining elevated pharyngeal oxygen concentrations, particularly when the ventilator FiO_2_ is set at 100%.

Evidence suggests that SJOV results in a large reduction in the risk of hypoxemia (SpO_2_ < 90%) and likely results in a large reduction in the risk of severe hypoxemia (SpO_2_ < 75%). The robustness of these findings is supported by evidence that SJOV may result in a large reduction in the need for jaw lift and mask ventilation. Consistency in the direction and magnitude of the effects across oxygenation and airway management outcomes provides strong evidence of the efficacy of SJOV in preventing hypoxemic events during procedural sedation. Although concerns about the methodological quality of the included studies resulted in low certainty of evidence for the primary outcome, TSA revealed that the evidence was sufficient to support the efficacy of SJOV in reducing the risk of hypoxemia during procedural sedation. This finding suggests that despite the limitations in the quality of the included trials, the observed treatment effect is likely to be reliable.

Regarding adverse events, the evidence from our meta-analysis indicated that there may be little to no difference in nasal bleeding risk between the SJOV and control groups. For hemodynamic instability (e.g., bradycardia), the evidence is very uncertain about any differences between SJOV and control due to the very low certainty of evidence. However, sensitivity analysis revealed that the removal of one study [33] resulted in a significantly higher risk of nasal bleeding in the SJOV group (*p* = 0.03), suggesting the need for further research to clarify this potential adverse event. Postprocedural sore throat was the only adverse event that probably occurred more frequently with SJOV, based on evidence of moderate certainty. The increased risk of sore throat in the SJOV group (RR = 1.71) may be attributed to the placement of the tube device in the supraglottic region and the high-pressure oxygen flow directed towards the vocal cords.

Notably, the certainty of the evidence varied across the outcomes evaluated in this meta-analysis. Findings related to severe hypoxemia, sore throat, and propofol dosage had moderate certainty of evidence, providing a higher level of confidence in these results. The reduction in severe hypoxemia and the absence of a difference in propofol dosage between the SJOV and control groups highlight that SJOV can effectively prevent severe desaturation events without necessitating a reduction in sedative doses. However, the increased risk of sore throat with SJOV should be considered and communicated to patients. In contrast, the outcomes of hypoxemia, subclinical respiratory depression, jaw thrust, mask ventilation, and nasal bleeding had low certainty of evidence, indicating that these findings should be interpreted with caution. Clinicians should weigh the potential advantages of SJOV against the uncertainty surrounding these outcomes when making decisions regarding its use in procedural sedation.

The development of barotrauma remains a concern when jet ventilation is used [51, 52]. Although no barotrauma was reported in the current meta-analysis, SJOV should be used judiciously or avoided in patients with bullous lung disease, pulmonary emphysema, or a history of spontaneous pneumothorax. In these patients, the high pressure required for adequate jet ventilation may overwhelm areas with cystic lung architecture and precipitate tension pneumothorax or pneumomediastinum. If SJOV is used as a rescue device in emphysematous or post-pneumothorax patients, the lowest effective jet ventilation pressure should be used, and chest radiography after the procedure should be considered to rule out the development of barotrauma.

Current evidence for the use of SJOV in difficult airway management is limited to observational studies and case reports [53–55]. Case reports observed improvements in oxygen saturation when SJOV was used in emergency “cannot intubate, cannot ventilate” scenarios [53, 54]. Although our meta-analysis demonstrated the efficacy and safety of SJOV in reducing the risk of hypoxemia during procedural sedation, we cannot draw definitive conclusions regarding the efficacy and safety of SJOV in patients with difficult airways. Future research should carefully investigate complications (e.g., sore throat and barotrauma), particularly in high-risk groups, to provide more robust recommendations for clinical practice.

One review article [30] suggests that SJOV can be used without a specific time constraint, but it acknowledges that the longest reported usage duration is 45 min in a patient with “cannot intubate and cannot ventilate” emergency difficult airway [53]. In our meta-analysis, the maximum duration noted in the included studies was 32 min. Therefore, the safety of prolonged SJOV use remains unclear. Based on available evidence, SJOV and HFNO [25, 27, 56] appear to be more effective than conventional oxygen therapy in reducing the risk of hypoxemia during sedative procedures. The current meta-analysis found that SJOV was associated with a 74% relative risk reduction in hypoxemia (RR = 0.26) compared to the control. Previous meta-analyses demonstrated that HFNO significantly reduced the risk of hypoxemia in sedated patients undergoing sedative procedures (RR, 0.23–0.37) [25, 27, 56]. Although direct comparisons between SJOV and HFNO are lacking, both techniques appear to offer substantial benefits in maintaining adequate oxygenation during procedural sedation. Nevertheless, SJOV may provide some degree of ventilatory support in addition to oxygenation, whereas HFNO primarily focuses on oxygenation optimization. Further head-to-head trials comparing SJOV and HFNO are warranted to determine whether one approach is superior to the other in terms of efficacy, safety, and cost.

This meta-analysis had several limitations. First, only one study enrolled patients with a high body mass index [49]. Given the risk of hypoxemia due to oxygen desaturation and hypoventilation in obese patients, further studies are required to examine the effects of SJOV in this vulnerable population. Second, considering the lack of heterogeneity in the primary outcome across studies, we did not perform subgroup analyses to examine whether the effects differed across different types of procedures. The efficacy and safety of SJOV in different patient subgroups should be examined in future studies. Third, all included RCTs were conducted in China, which may affect the generalizability of the results to other geographic settings and ethnicities. Fourth, the potential for publication bias remains for several secondary outcomes, which may be partially attributable to the fact that we did not contact authors for unpublished data and excluded conference abstracts from our analysis. While these decisions were made to ensure that all included studies could be adequately assessed for methodological quality, they may have inadvertently contributed to publication bias by omitting relevant unpublished or preliminary findings. Finally, the reduction in hypoxemia risk does not equate to improved clinical outcomes. Future studies should determine whether improved intraprocedural oxygenation with SJOV leads to short recovery times, low healthcare costs, and other patient-centered outcomes.

## Conclusion

This meta-analysis of 12 RCTs demonstrated that SJOV likely resulted in a large reduction in the risk of severe hypoxemia and probably resulted in little to no difference in sedative doses between the groups. However, SJOV is probably associated with an increased risk of sore throat, while showing little to no difference in nasal bleeding. Moreover, some complications (e.g., barotrauma) have not yet been evaluated, necessitating careful investigation of adverse events in future studies. The safety and efficacy of prolonged SJOV use in patients with difficult airways remain unclear. Clinicians should carefully balance potential benefits and risks for individual patients, use the lowest effective ventilation pressure if SJOV is employed, and vigilantly monitor for adverse events.

## Supplementary Information

Additional file 1: Supplemental Figs. 1–16

Additional file 2: Supplemental Tables 1–5

## Data Availability

The datasets used and/or analyzed in the current study are available from the corresponding author upon reasonable request.
