# Supplementary material for: Efficacy and safety of supraglottic jet oxygenation and ventilation to minimize sedation-related hypoxemia: a meta-analysis with GRADE approach
Source: Syst Rev. 2024 Nov 14;13:281. doi: 10.1186/s13643-024-02707-w (PMC11566649; doi:10.1186/s13643-024-02707-w)

**Supplemental Figure**

**Supplemental Figure 1.** Forest plot showing the risk of nasal bleeding in supraglottic jet oxygenation and ventilation (SJOV) versus control group. NPA: nasopharyngeal airway; WNJ: Wei nasal jet tube; NC: nasal cannula; M: mask.


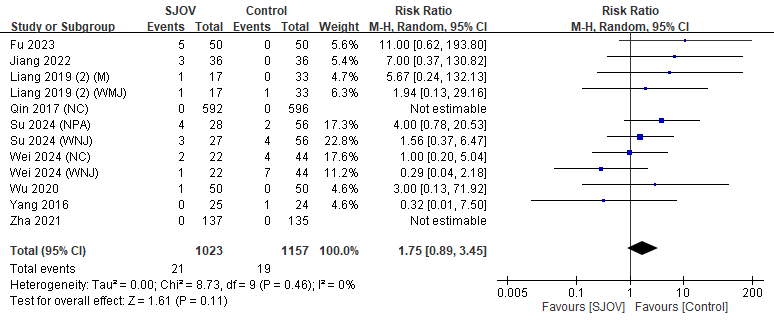


**Supplemental Figure 2.** Forest plot showing the risk of sore throat in supraglottic jet oxygenation and ventilation (SJOV) versus control group. NPA: nasopharyngeal airway; WNJ: Wei nasal jet tube; NC: nasal cannula; M: mask.


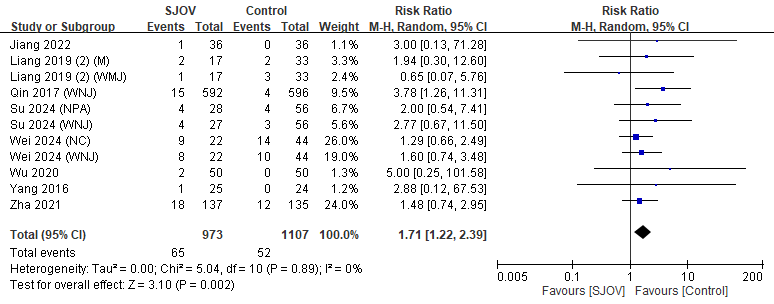


**Supplemental Figure 3.** Forest plot showing the risk of bradycardia in supraglottic jet oxygenation and ventilation (SJOV) versus control group. NPA: nasopharyngeal airway; WNJ: Wei nasal jet tube; NC: nasal cannula; M: mask.


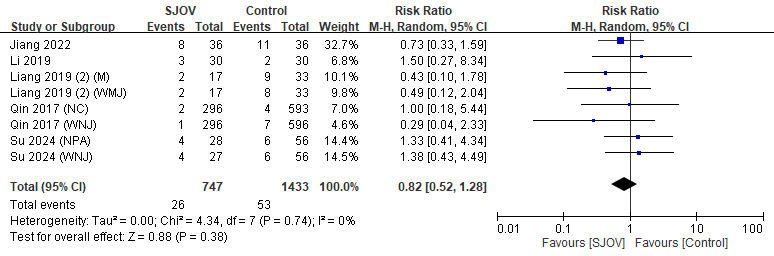


**Supplemental Figure 4.** Forest plot showing the risk of tachycardia in supraglottic jet oxygenation and ventilation (SJOV) versus control group. NPA: nasopharyngeal airway; WNJ: Wei nasal jet tube; NC: nasal cannula; M: mask.


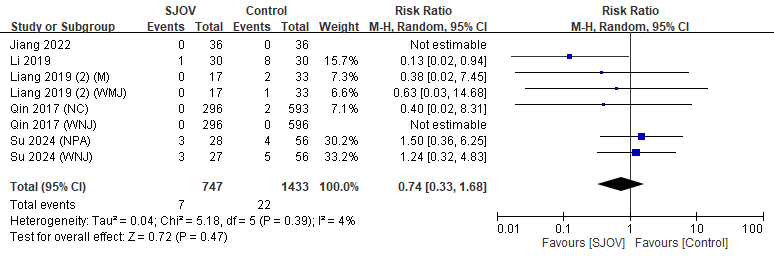


**Supplemental Figure 5.** Forest plot showing the risk of hypertension in supraglottic jet oxygenation and ventilation (SJOV) versus control group. NPA: nasopharyngeal airway; WNJ: Wei nasal jet tube; NC: nasal cannula; M: mask.

**
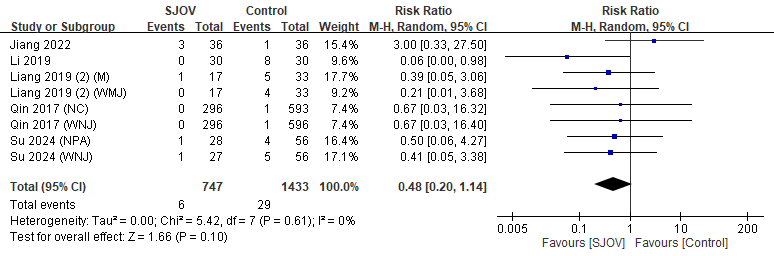
**

**Supplemental Figure 6.** Forest plot showing the risk of hypotension in supraglottic jet oxygenation and ventilation (SJOV) versus control group. NPA: nasopharyngeal airway; WNJ: Wei nasal jet tube; NC: nasal cannula; M: mask.


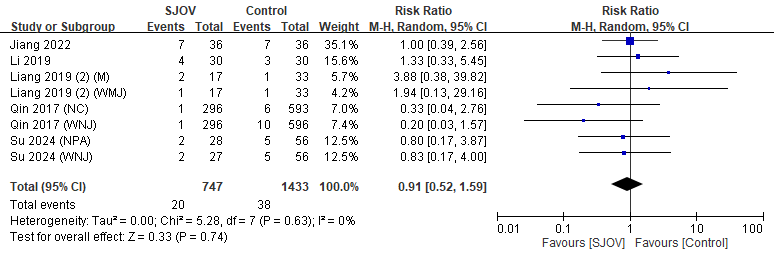


**Supplemental Figure 7.** Forest plot showing the difference in dosage of propofol in supraglottic jet oxygenation and ventilation (SJOV) versus control group. NPA: nasopharyngeal airway; WNJ: Wei nasal jet tube; NC: nasal cannula; M: mask.

**
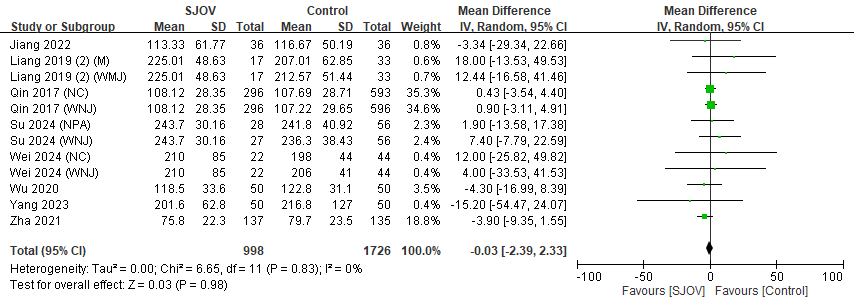
**

**Supplemental Figure 8.** Forest plot showing the difference in procedural time in supraglottic jet oxygenation and ventilation (SJOV) versus control group. NPA: nasopharyngeal airway; WNJ: Wei nasal jet tube; NC: nasal cannula.


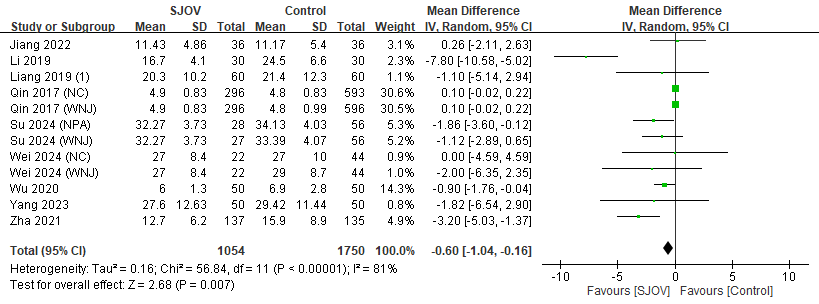


**Supplemental Figure 9.** Funnel plot for hypoxemia


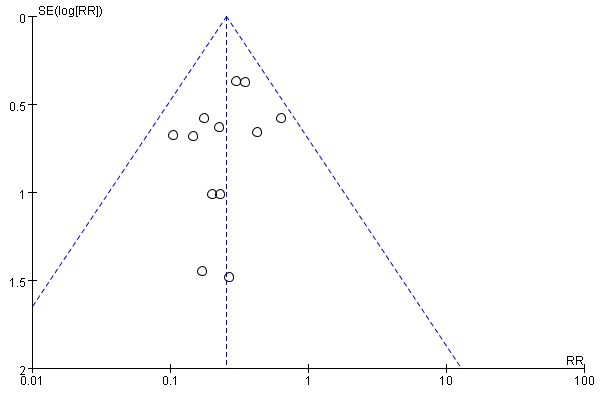


**Supplemental Figure 10.** Funnel plot for Subclinical respiratory depression


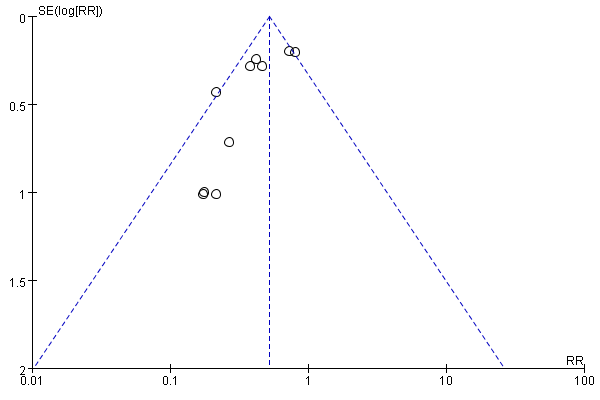


**Supplemental Figure 11.** Funnel plot for jaw thrust


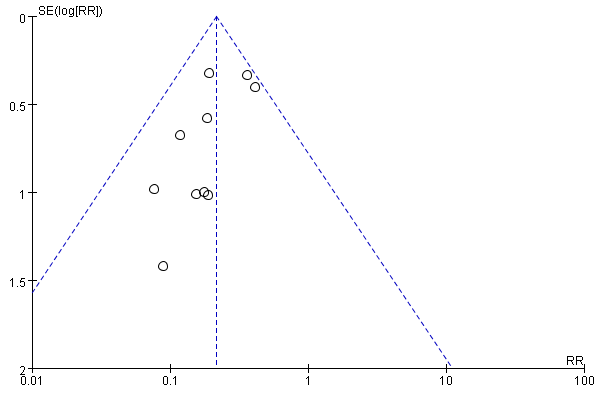


**Supplemental Figure 12.** Funnel plot for mask ventilation


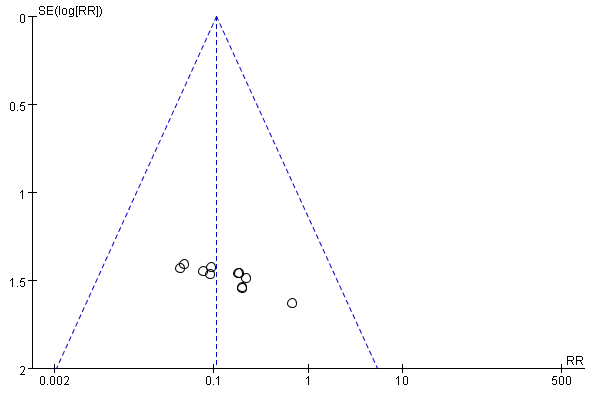


**Supplemental Figure 13.** Funnel plot for nasal bleeding


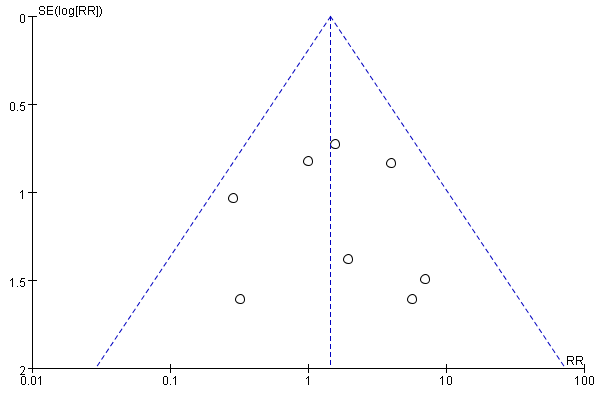


**Supplemental Figure 14.** Funnel plot for sore throat


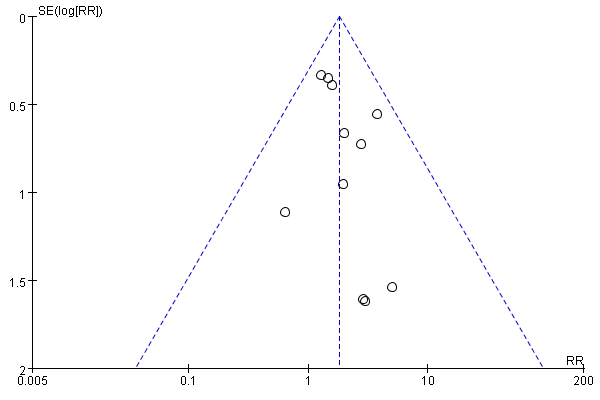


**Supplemental Figure 15** Funnel plot for dosage of propofol


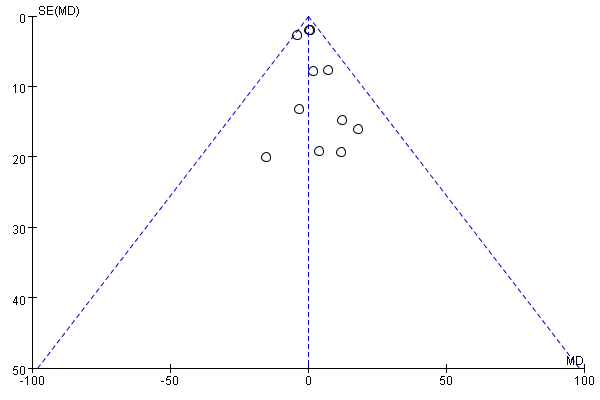


**Supplemental Figure 16.** Funnel plot for procedural time


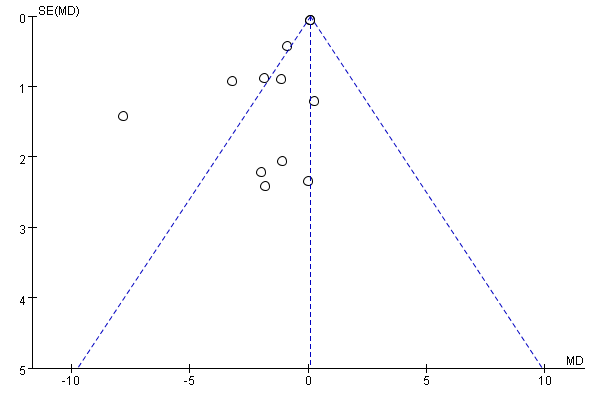

Supplement: Supplementary file 1 — Additional file 1: Supplemental Figs. 1–16 [file 13643_2024_2707_MOESM1_ESM.docx]
