# Supplementary material for: Efficacy and safety of supraglottic jet oxygenation and ventilation to minimize sedation-related hypoxemia: a meta-analysis with GRADE approach
Source: Syst Rev. 2024 Nov 14;13:281. doi: 10.1186/s13643-024-02707-w (PMC11566649; doi:10.1186/s13643-024-02707-w)
Supplement: Supplementary file 2 — Additional file 2: Supplemental Tables 1–5 [file 13643_2024_2707_MOESM2_ESM.docx]

**Supplemental Table 1.** Search strategies for Medline

| 1 | ("Sedation" or "Sedative procedure" or "Procedural sedation" or "Monitored anesthesia care" or "Colonoscopy" or "Bronchoscopy" or "Endoscopic retrograde cholangiopancreatography" or "gastrointestinal endoscopy" or "gastroscopy" or "propofol" or "intravenous anesthesia").mp. |
| --- | --- |
| 2 | exp "Deep Sedation"/ |
| 3 | ("Supraglottic jet oxygenation" or "SJOV" or "Wei nasal jet tube" or "supraglottic jet oxygenation and ventilation" or "Transnasal jet ventilation" or "Wei nasal jet ventilation" or “jet ventilation” or "apneic ventilation" or "high frequency jet ventilation").mp. |
| 4 | ("Respiratory depression" or "Hypoxemia" or "Nasal bleeding" or "Hypoxia" or "Adverse events" or "tracheal intubation").mp. |
| 5 | (1 or 2) and 3 and 4 |
| 6 | 5 and (((randomized controlled trial or controlled clinical trial).pt. or randomi*ed.ab. or placebo.ab. or drug therapy.fs. or randomly.ab. or trial.ab. or groups.ab.) not (exp animals/ not humans.sh.)) |

**Supplemental table 2.** Studies excluded after full texts assessed

| Reason for exclusion: Lack of control group |
| --- |
| 1. Anwar, M., Fritze, R., Base, E., Wasserscheid, T., Wolfram, N., Koinig, H., Errhalt, P. (2020). Infraglottic versus supraglottic jet-ventilation for endobronchial ultrasound-guided transbronchial needle aspiration: A randomised controlled trial. *European Journal of Anaesthesiology\| EJA, 37*(11), 999-1007. 2. Leiter, R., Aliverti, A., Priori, R., Staun, P., Lo Mauro, A., Larsson, A., & Frykholm, P. (2012). Comparison of superimposed high-frequency jet ventilation with conventional jet ventilation for laryngeal surgery. *British Journal of Anaesthesia, 108*(4), 690-697. 3. Huang X.Y., Hu T., Liu Y.J. (2023). Comparison of Three Different Jet Ventilation Modes in Painless Fiberoptic Bronchoscopy. Chinese Medical Innovation, 20(19), 625. |
| Reason for exclusion: Tracheal intubation or laryngeal mask airway involvement |
| 1. Supraglottic jet oxygenation and ventilation-assisted fibre-optic bronchoscope intubation in patients with difficult airways. (2017). Internal and emergency medicine, 12(5), 667‐673. 2. Boyce, J. R., Waite, P. D., Louis, P. J., & Ness, T. J. (2003). Transnasal jet ventilation is a useful adjunct to teach fibreoptic intubation: a preliminary report. Canadian Journal of Anaesthesia, 50(10), 1056-1060. 3. Ihar, G., Hieber, C., Schaberning, C., Karincuk, P., Adel, S., Plöchl, W., & Aloy, A. (1999). Supralaryngeal tubeless combined high-frequency jet ventilation for laser surgery of the larynx and trachea. British Journal of Anaesthesia, 83(6), 940-942. 4. Rezaie-Majd, A., Bigenzahn, W., Denk, D.-M., Burian, M., Kornfehl, J., Grasl, M. C., . . . Aloy, A. (2006). Superimposed high-frequency jet ventilation (SHFJV) for endoscopic laryngotracheal surgery in more than 1500 patients. BJA: British Journal of Anaesthesia, 96(5), 650-659. 5. Wu, C., Wei, J., Cen, Q., Sha, X., Cai, Q., Ma, W., & Cao, Y. (2017). Supraglottic jet oxygenation and ventilation-assisted fibre-optic bronchoscope intubation in patients with difficult airways. Internal & Emergency Medicine, 12(5), 667-673. doi:https://dx.doi.org/10.1007/s11739-016-1531-6 6. Yang, M., Wang, B., Hou, Q., Zhou, Y., Li, N., Wang, H., . . . Cheng, Q. (2021). High frequency jet ventilation through mask contributes to oxygen therapy among patients undergoing bronchoscopic intervention under deep sedation. BMC Anesthesiology, 21(1), 1-9. 7. Wu L. (2021). Application of Wei Nasal Jet Airway Ventilation in Obese Patients Undergoing Hysteroscopy. Jiangsu Medical Journal, 47(6), 623. 8. Zhuang H., Zhu Y., Zhuang M., Shi Z. (2020). Application of Wei Nasal Jet Airway Ventilation in Patients with Laryngeal Disease Before Endotracheal Intubation. *Anhui Medical Journal*, 41(7), 764. 9. Li J.R., Wu Q.H., Lin C.L., Shao Y.H. (2019). Ventilation Effects of High-Frequency Jet Ventilation in Non-Intubated General Anesthesia for Treating Hyperhidrosis. *Journal of Hainan Medical University*, 25(07), 506. DOI: 10.13210/j.cnki.jhmu.20190225.002 10. Zha B.J., Wu Z.Y., Hu J., Xie P., Li Q.Y. (2022). Effects of Laryngeal Jet Ventilation in General Anesthesia Applied by Non-Anesthesiology Residents. Guangxi Medical Journal, 44(3), 258. 11. Zhang X.J., Yao H.B., Lu F., Xu W.C., Li J.H., Li T., Lei J.Y., Wang S.Q. (2021). Study on the Application Effect of Endotracheal Catheter-Guided Laryngeal Jet Ventilation in Patients with Difficult Airways Undergoing Painless Gastroscopy. *Jiangxi Medical Journal*, 56(5), 574. 12. Zha B.J., Xie P., Hu J., Xiong H.P. (2021). Application Study of Laryngeal Jet Ventilation in Standardized Training. Jiangxi Medical Journal, 56(7), 938. 13. Zhang M.J., Jia Q.S., Wu L.N. (2023). Comparison of the Impact of Different Ventilation Methods on Stress Response Levels in High-Risk Patients with Difficult Airways During Painless Gastroscopy. *Liaoning Medical Journal*, 37(5), 112. |
| Reason for exclusion: absence of an intervention group |
| 1. Shao, L. J., Hong, F. X., Liu, F. K., Wan, L., & Xue, F. S. (2021). Prospective, randomized comparison of two supplemental oxygen methods during gastroscopy with propofol mono-sedation in obese patients. World Journal of Clinical Cases, 9(20), 5479-5489. doi:https://dx.doi.org/10.12998/wjcc.v9.i20.5479 2. Tan, H., Wan, L., Liu, F., Xue, F., & Shao, L. (2022). Application of Wei nasal jet tube in painless gastroscopy in patients over age 60. Chinese Journal of Digestive Endoscopy, 39(9), 735‐738. doi:10.3760/cma.j.cn321463-20220412-00198 3. Qiu L.J., Wan Y., Liu X.B., Hong F.X., Han F.S. (2021). Effects of Wei Nasal Jet Airway and Nasal Catheter Oxygen Inhalation on Hemodynamics in Patients Undergoing Painless Gastroscopy. Chinese Practical Diagnosis and Therapy, 35(6), 610. |

**Supplemental table 3.** Other outcomes

| **Studies** | **Barotrauma** | **CO_2_ measurement (SJVO vs. control)** | **Estimated stomach volume (mL)(before-after)** |
| --- | --- | --- | --- |
| Fu 2023 | No mention | PETCO2: 39.3±0.9 vs. 42.5±2 (***p* < 0.001**) | Not reported |
| Jiang 2022 | No barotrauma | Not reported | Not reported |
| Li 2019 | No mention | 40.8±4.2 vs. 39.6±4.6 (***p* = 0.41**) | Not reported |
| Liang 2019 (1) | No mention | Not reported | 18. 8 vs. 18. 9 |
| Liang 2019 (2) | No barotrauma | PETCO2 < 10 mmHg SJOV: 9% vs. conventional oxygenation: 36%-33%; **(*p* < 0.05)** | 18.78 vs 18.89 |
| Qin 2017 | No barotrauma | Not reported | Not reported |
| Su 2024 | No barotrauma | Not reported | Not reported |
| Wei 2024 | No barotrauma | Not reported | Not reported |
| Wu 2020 | No barotrauma | Not reported | Not reported |
| Yang 2023 | No mention | ABG 56.36±11.29 vs. 63.10±12.44 (***p* = 0.006**) | Not reported |
| Yang 2016 | No barotrauma | PetCO2 41.2±3.7 vs. 41.5±4.3 (***p* = 0.87**) | Not reported |
| Zha 2021 | No barotrauma | Not reported | Not reported |

**Supplemental Table 4.** The funding sources for each study

| Fu 2023 | Supported by the National Natural Science Foundation of China (82100652) |
| --- | --- |
| Jiang 2022 | This work was supported by the Group-aiding Tibet Medical Program of the Tibet Autonomous Region Natural Science Foundation [grant number XZ2022ZR-ZY11(Z)]. |
| Li 2019 | Supported by Putian Science and Technology Fund (No. 2018S3Y006) |
| Liang 2019 (1) | na |
| Liang 2019 (2) | no funding |
| Qin 2017 | National Natural Science Foundation of China (nos 81271220 and 81571030); Major State Basic Research Development Research Programme of China (973 program, no. 2013CB531902); Shanghai Municipal Commission of Health and Family Planning Founding for Key Developing Disciplines (no. 2015ZB0101); Shanghai Pudong New Area Science and Technology Commission Funding (PW2016D-4). |
| Su 2024 | Shanghai East Hospital Affiliated to Tongji University Scientific Research Foundation (Grant no. DFRC2021008); Shanghai Association of Chinese Integrative Medicine Research Foundation (Grant no. MT23-2). |
| Wei 2024 | This work was supported by grants from Shanghai Hospital Development Centre (no. SHDC2020CR3044B), Shanghai Municipal Health Commission (no. 202040004), and Development Fund for the Department of Anesthesiology, Shanghai Pulmonary Hospital. |
| Wu 2020 | Supported by the Quanzhou Science and Technology Program (2016Z066) |
| Yang 2023 | Dr. Huafeng Wei is the inventor of the Wei Nasal Jet Tube (Wei Nasal Jet or WNJ), which was used to generate SJOV in this study |
| Yang 2016 | This work was supported by Shanghai Health Bureau Foundation (No: SH20114Y147). |
| Zha 2021 | no funding |

na: not available

**Supplemental Table 5.** The risk of bias assessment for the secondary outcomes

| **Outcomes:** **Subclinical respiratory depression** | | | | | | |
| --- | --- | --- | --- | --- | --- | --- |
| **Studies** | **D1** | **D2** | **D3** | **D4** | **D5** | **Overall risk of bias** |
| Fu 2023 | Some Concerns | Low | Low | Low | Low | Some Concerns |
| Jiang 2022 | Some Concerns | Low | Low | Low | Low | Some Concerns |
| Liang 2019 (1) | Some Concerns | Low | Low | Low | Low | Some Concerns |
| Liang 2019 (2) | Some Concerns | Low | Low | Low | Low | Some Concerns |
| Qin 2017 | Some Concerns | Low | Low | Low | Low | Some Concerns |
| Su 2024 | Some Concerns | Low | Low | Low | Low | Some Concerns |
| Wei 2024 | Some Concerns | Low | Low | Low | Low | Some Concerns |
| Wu 2020 | Some Concerns | Low | Low | Low | Low | Some Concerns |
| Zha 2021 | Some Concerns | Low | Low | Low | Low | Some Concerns |
| **Outcomes:** **Severe hypoxemia** | | | | | | |
| **Studies** | **D1** | **D2** | **D3** | **D4** | **D5** | **Overall risk of bias** |
| Fu 2023 | Some Concerns | Low | Low | Low | Low | Some Concerns |
| Jiang 2022 | Some Concerns | Low | Low | Low | Low | Some Concerns |
| Qin 2017 | Some Concerns | Low | Low | Low | Low | Some Concerns |
| Su 2024 | Some Concerns | Low | Low | Low | Low | Some Concerns |
| Wei 2024 | Some Concerns | Low | Low | Low | Low | Some Concerns |
| Zha 2021 | Some Concerns | Low | Low | Low | Low | Some Concerns |
| **Outcomes:** **Jaw-thrust** | | | | | | |
| **Studies** | **D1** | **D2** | **D3** | **D4** | **D5** | **Overall risk of bias** |
| Fu 2023 | Some Concerns | Low | Low | Low | Low | Some Concerns |
| Jiang 2022 | Some Concerns | Low | Low | Low | Low | Some Concerns |
| Liang 2019 (1) | Some Concerns | Low | Low | Low | Low | Some Concerns |
| Liang 2019 (2) | Some Concerns | Low | Low | Low | Low | Some Concerns |
| Qin 2017 | Some Concerns | Low | Low | Low | Low | Some Concerns |
| Su 2024 | Some Concerns | Low | Low | Low | Low | Some Concerns |
| Wei 2024 | Some Concerns | Low | Low | Low | Low | Some Concerns |
| Wu 2020 | Some Concerns | Low | Low | Low | Low | Some Concerns |
| Zha 2021 | Some Concerns | Low | Low | Low | Low | Some Concerns |
| **Outcomes:** **Mask ventilation** | | | | | | |
| **Studies** | **D1** | **D2** | **D3** | **D4** | **D5** | **Overall risk of bias** |
| Fu 2023 | Some Concerns | Low | Low | Low | Low | Some Concerns |
| Jiang 2022 | Some Concerns | Low | Low | Low | Low | Some Concerns |
| Liang 2019 (1) | Some Concerns | Low | Low | Low | Low | Some Concerns |
| Qin 2017 | Some Concerns | Low | Low | Low | Low | Some Concerns |
| Su 2024 | Some Concerns | Low | Low | Low | Low | Some Concerns |
| Wei 2024 | Some Concerns | Low | Low | Low | Low | Some Concerns |
| Wu 2020 | Some Concerns | Low | Low | Low | Low | Some Concerns |
| Zha 2021 | Some Concerns | Low | Low | Low | Low | Some Concerns |
| **Outcome: Nasal bleeding** | | | | | | |
| **Studies** | **D1** | **D2** | **D3** | **D4** | **D5** | **Overall risk of bias** |
| Fu 2023 | Some Concerns | Low | Low | Low | Low | Some Concerns |
| Jiang 2022 | Some Concerns | Low | Low | Low | Low | Some Concerns |
| Liang 2019 (2) | Some Concerns | Low | Low | Low | Low | Some Concerns |
| Qin 2017 | Some Concerns | Low | Low | Low | Low | Some Concerns |
| Su 2024 | Some Concerns | Low | Low | Low | Low | Some Concerns |
| Wei 2024 | Some Concerns | Low | Low | Low | Low | Some Concerns |
| Wu 2020 | Some Concerns | Low | Low | Low | Low | Some Concerns |
| Yang 2016 | Some Concerns | Low | Low | Low | Low | Some Concerns |
| Zha 2021 | Some Concerns | Low | Low | Low | Low | Some Concerns |
| **Outcome: Sore throat** | | | | | | |
| **Studies** | **D1** | **D2** | **D3** | **D4** | **D5** | **Overall risk of bias** |
| Jiang 2022 | Some Concerns | Low | Low | Low | Low | Some Concerns |
| Liang 2019 (2) | Some Concerns | Low | Low | Low | Low | Some Concerns |
| Qin 2017 | Some Concerns | Low | Low | Low | Low | Some Concerns |
| Su 2024 | Some Concerns | Low | Low | Low | Low | Some Concerns |
| Wei 2024 | Some Concerns | Low | Low | Low | Low | Some Concerns |
| Wu 2020 | Some Concerns | Low | Low | Low | Low | Some Concerns |
| Yang 2016 | Some Concerns | Low | Low | Low | Low | Some Concerns |
| Zha 2021 | Some Concerns | Low | Low | Low | Low | Some Concerns |
| **Outcome: Bradycardia** | | | | | | |
| **Studies** | **D1** | **D2** | **D3** | **D4** | **D5** | **Overall risk of bias** |
| Jiang 2022 | Some Concerns | Low | Low | Low | Low | Some Concerns |
| Li 2019 | Some Concerns | Low | Low | Low | Some Concerns | High |
| Liang 2019 (2) | Some Concerns | Low | Low | Low | Low | Some Concerns |
| Qin 2017 | Some Concerns | Low | Low | Low | Low | Some Concerns |
| Su 2024 | Some Concerns | Low | Low | Low | Low | Some Concerns |
| **Outcome: Tachycardia** | | | | | | |
| **Studies** | **D1** | **D2** | **D3** | **D4** | **D5** | **Overall risk of bias** |
| Jiang 2022 | Some Concerns | Low | Low | Low | Low | Some Concerns |
| Li 2019 | Some Concerns | Low | Low | Low | Some Concerns | High |
| Liang 2019 (2) | Some Concerns | Low | Low | Low | Low | Some Concerns |
| Qin 2017 | Some Concerns | Low | Low | Low | Low | Some Concerns |
| Su 2024 | Some Concerns | Low | Low | Low | Low | Some Concerns |
| **Outcome: Hypertension** | | | | | | |
| **Studies** | **D1** | **D2** | **D3** | **D4** | **D5** | **Overall risk of bias** |
| Jiang 2022 | Some Concerns | Low | Low | Low | Low | Some Concerns |
| Li 2019 | Some Concerns | Low | Low | Low | Some Concerns | High |
| Liang 2019 (2) | Some Concerns | Low | Low | Low | Low | Some Concerns |
| Qin 2017 | Some Concerns | Low | Low | Low | Low | Some Concerns |
| Su 2024 | Some Concerns | Low | Low | Low | Low | Some Concerns |
| **Outcome: Hypotension** | | | | | | |
| **Studies** | **D1** | **D2** | **D3** | **D4** | **D5** | **Overall risk of bias** |
| Jiang 2022 | Some Concerns | Low | Low | Low | Low | Some Concerns |
| Li 2019 | Some Concerns | Low | Low | Low | Some Concerns | High |
| Liang 2019 (2) | Some Concerns | Low | Low | Low | Low | Some Concerns |
| Qin 2017 | Some Concerns | Low | Low | Low | Low | Some Concerns |
| Su 2024 | Some Concerns | Low | Low | Low | Low | Some Concerns |
| **Outcome: Dosage of propofol** | | | | | | |
| **Studies** | **D1** | **D2** | **D3** | **D4** | **D5** | **Overall risk of bias** |
| Jiang 2022 | Some Concerns | Low | Low | Low | Low | Some Concerns |
| Liang 2019 (2) | Some Concerns | Low | Low | Low | Low | Some Concerns |
| Qin 2017 | Some Concerns | Low | Low | Low | Low | Some Concerns |
| Su 2024 | Some Concerns | Low | Low | Low | Low | Some Concerns |
| Wei 2024 | Some Concerns | Low | Low | Low | Low | Some Concerns |
| Wu 2020 | Some Concerns | Low | Low | Low | Low | Some Concerns |
| Yang 2023 | Low | Low | Low | Low | Low | Some Concerns |
| Zha 2021 | Some Concerns | Low | Low | Low | Low | Some Concerns |
| **Outcome: Procedural time** | | | | | | |
| **Studies** | **D1** | **D2** | **D3** | **D4** | **D5** | **Overall risk of bias** |
| Jiang 2022 | Some Concerns | Low | Low | Low | Low | Some Concerns |
| Li 2019 | Some Concerns | Low | Low | Low | Some Concerns | High |
| Liang 2019 (1) | Some Concerns | Low | Low | Low | Low | Some Concerns |
| Qin 2017 | Some Concerns | Low | Low | Low | Low | Some Concerns |
| Su 2024 | Some Concerns | Low | Low | Low | Low | Some Concerns |
| Wei 2024 | Some Concerns | Low | Low | Low | Low | Some Concerns |
| Wu 2020 | Some Concerns | Low | Low | Low | Low | Some Concerns |
| Yang 2023 | Low | Low | Low | Low | Low | Some Concerns |
| Zha 2021 | Some Concerns | Low | Low | Low | Low | Some Concerns |

Domains:

D1: Bias arising from the randomization process.

D2: Bias due to deviations from intended intervention.

D3: Bias due to missing outcome data.

D4: Bias in measurement of the outcome.

D5: Bias in selection of the reported result.
